# Gambling related family coping and the impact of problem gambling on families in Hong Kong

**DOI:** 10.1186/s40405-016-0009-9

**Published:** 2016-03-31

**Authors:** Elda Mei Lo Chan, Nicki A. Dowling, Alun C. Jackson, Daniel Tan-lei Shek

**Affiliations:** 1 Integrated Centre on Addiction Prevention and Treatment, Tung Wah Group of Hospitals, Hong Kong SAR, China; 2 School of Psychology, Faculty of Health, Deakin University, Melbourne, Australia; 3 Melbourne Graduate School of Education, University of Melbourne, Melbourne, Australia; 4 Centre on Behavioural Health, University of Hong Kong, Hong Kong SAR, China; 5 Department of Applied Social Sciences, The Hong Kong Polytechnic University, Hong Kong SAR, China

**Keywords:** Family coping, Impact of problem gambling, Families, Chinese, Hong Kong

## Abstract

Despite substantial evidence that problem gambling is associated with a wide range of family difficulties, limited effort has been devoted to studying the negative impacts on family members as a result of problem gambling and how they cope and function under the impacts of problem gambling in Chinese communities. Among the very few Chinese-specific gambling-related family impact studies, none have examined how gambling-related family coping responses are related to gambling-related family impacts. Based on a sample of treatment-seeking Chinese family members of problem gamblers, this study aimed to explore: (1) the demographic characteristics and health and psychological well-being of the family members; (2) the gambling-related family member impacts (active disturbance, worrying behavior); (3) the family coping strategies (engaged, tolerant-inactive and withdrawal coping); (4) the relationship between gambling-related family member impacts, psychological distress and family coping strategies. It was hypothesized that positive significant relationships would be found between family member impacts, psychological distress and family coping strategies. From March 2011 to February 2012, a total of 103 family members of problem gamblers who sought help from Tung Wah Group of Hospitals Even Centre in Hong Kong were interviewed. Results showed that a majority of family members were partners or ex-partners of the gambler with low or no income. A large proportion of participants reported moderate to high psychological distress (72.6 %), poor to fair general health (60.2 %), and poor to neither good nor bad quality of life (61.1 %). Family member impacts were positively significantly correlated to all family coping strategies and psychological distress. Tolerant-inactive coping had the strongest relationships with family member impacts and psychological distress. Strong relationships between family member impacts and psychological distress were also found. The results provide preliminary support for aspects of the stress–strain–coping–support model in the Chinese culture. It is suggested that family member-specific treatment groups targeting family coping are required to alleviate the level of negative impacts of gambling disorder on family members.

## Background and rationale

Although growth of the gambling industry can contribute the economy in many jurisdictions, adverse impacts as a result of problem gambling such as disrupted family and marital relationships, family dysfunction, family financial difficulties, mental and physical health problems and diminished life fulfilment have been reported (Dowling, Smith & Thomas, 2007; Dowling, Jackson, Suomi et al., 2014a; Dowling, Rodda, Lubman & Jackson, 2014b; Dowling, Suomi, Jackson & Lavis, 2015; Hodgins, Shead & Makarchuk, 2007; Kalischuk, Novatzki, Cardwell, Klein & Solowoniuk, 2006; Suomi, Jackson, Dowling, et al., 2013).

Extensive studies have been conducted on the impact of gambling on family members including the spouse, children, parents, grandparents, siblings and other extended family members. For example, Dowling et al. (2014a) studied a group of 366 concerned significant others (CSOs) accessing an on-line gambling counselling service. The findings revealed that most of the CSOs were female intimate partners of problem gamblers under 30 years old. The most commonly reported adverse impacts experienced by these CSOs included emotional distress and impacts on the relationship, followed by impacts on social life and finances. Some family members also reported impacts on employment and physical health. There is also evidence that the family members of problem gamblers are at a higher risk of having alcohol disorder, substance use disorder, and physical and mental health problems (Black, Monahan, Temkit & Shaw, 2006; Orford, Velleman, Natera, Templeton & Copello, 2013; Wenzel, Oren & Bakken, 2009). Other studies have revealed that family violence, including intimate partner and child abuse, is a serious adverse effect of problem gambling (Dowling et al., 2014a, b; Suomi et al., 2013).

Understanding how families function and cope in such situations of adversity is clearly a crucial topic in the development of a family sensitive and effective treatment model. To gain in-depth understanding and generate insights into the development of a structured measure of family coping strategies, Orford, Rigby, Miller et al., (1992) conducted a qualitative study with 50 close relatives of people with drug problems in order to explore the structure of family coping responses based on the stress–strain–coping–support perspective. This perspective argues that the chronic stress of having an addiction in the family (conceptualised as active disturbance and worrying behaviour) results in strain experienced by family members in the form of some departure from a state of health and wellbeing and that the ways family members cope and the social support they receive influence the stress–strain relationship. From the qualitative data, they proposed and grouped the coping responses into eight categories: emotional, inactive, avoidant, tolerant, supportive to the user, controlling, confrontative and independent.

Orford, Natera, Divies et al., (1998) then advanced the understanding of ways of coping with addictive behaviour in the family. They further tested and developed the three broad coping responses: engaging, tolerating and withdrawing. The first coping response, engaging, has distinct characteristics. For example, one form of engagement can be more emotional and controlling; another can be more supportive and controlling; while another can be more assertive. It was argued that although different forms of engaged coping might be viewed independently, it would be difficult to separate them in a practical situation. Qualitative information gathered from the same study also reported a combination of characteristics of engaged coping response adopted by family members. The second response, tolerance, has a broad spectrum of characteristics: inactive, accepting, sacrificing and supporting. The third response is withdrawing: withdrawal from interaction with the relative with addiction. It has also been argued that the three responses are not distinct. Family members often reported coping in a multifaceted style when dealing with stress arising from addictive behaviour. In practical situations, family members often adopt a combination of coping responses and tend to select particular responses at different stages. This three factor typology of family coping responses was further tested and confirmed by Orford et al., (1998); Orford, Templeton, Velleman & Copello, (2005) as a reliable measure to examine family coping responses for family members who were affected by their relatives’ addictive behaviour.

Krishnan & Orford, (2002) subsequently applied the stress–strain–coping–support perspective to examine the impact of problem gambling on the family and the ways family members cope with the adverse impact. Sixteen close family members of gamblers were investigated using semi-structured interviews. The results revealed that most family members employed considerable “engaging” (specifically controlling) ways of coping with relatives’ gambling problems compared to the use of such responses by relatives of people with alcohol or drug problems. Reports from family members confirmed previous research findings that relatives of people with addiction problems often feel unsupported emotionally. The authors confirmed that the stress–strain–coping–support perspective, previously applied to families with alcohol and drug problems, also offered an appropriate framework for understanding how family members cope under the impact of their relatives’ problem gambling. Furthermore, when Orford et al. (2005) elaborated on the stress–strain–coping model involving family members affected by problem gambling, it was found that family member impacts (active disturbance and worrying behaviour) were significantly correlated with all family coping styles.

To date, however, most impact and coping research has been predominantly conducted in the West. There have been very few studies on the impact of problem gambling on families or the ways in which families cope in different indigenous Chinese cultural contexts (Breakthrough, 2002; Wong, Leung & Lau, 2009; Hsu, Lam & Wong, 2014). One study (Breakthrough, 2002) in Hong Kong found that 11.9 % of 1418 responses to a telephone survey reported adverse effects to some degree on the family members of problem gamblers. The impacts of gambling on the family included poorer psychological health and inter-personal relationships, and financial problems, such as having to sell valuable family items to repay gambling debts. Over 10.5 % of family members reported becoming agitated and irritable, experiencing a lack of communication and frequently engaging in heated arguments because of extreme worry. Family members of problem gamblers also reported being less optimistic about their future, having lower self-esteem and having no explicit goals in life compared to the non-problem gambling family members surveyed. Another study (Sobrun-Maharaj, Rossen & Wong 2013) revealed that adverse effects resulting from problem gambling among the Asian families and communities in New Zealand included social disconnection, family conflict such as aggression and violence towards family members, financial insecurity, and physical and mental health problems such as anxiety, anger, depression and insomnia. Studies examining how family coping strategies relate to these gambling related family impacts in Chinese communities are, however, non-existent.

## Aims and hypotheses

Research findings on family impacts and coping in Chinese communities will provide useful information to assist the formulation of a family-sensitive and culturally relevant treatment model which is essential to reduce the harmful effects of problem gambling and enhance family members’ coping responses in such stressful circumstances (Orford et al., 2013). However, family members who are affected by problem gambling often receive inadequate attention and assistance to deal with the impact of problem gambling. Studies have reported that when family members undergo treatment, there is an improvement in overall adjustment, better treatment outcomes and co-creation of life pathways for gambling individuals and their family members (Hodgins et al., 2007; Ingle, Marotta, McMillan & Wisdom, 2008; Kalischuk, 2010).

Based on a sample of treatment-seeking Chinese family members of problem gamblers, this study aimed to explore: (1) the demographic characteristics and health and psychological well-being of the family members; (2) the gambling-related family member impacts (active disturbance, worrying behavior); (3) the family coping strategies (engaged, tolerant-inactive and withdrawal coping); and (4) the relationships between gambling-related family member impacts (stress), psychological distress (strain) and family coping strategies (coping). It was hypothesized that positive significant relationships would be found between gambling-related family member impacts, psychological distress and family coping strategies.

## Method

### Sample and procedure

Data were collected from a Chinese dominant community. A purposeful sampling strategy was used to recruit family members who were negatively affected by gambling problems. Family members of gamblers who sought help from the Tung Wah Group of Hospitals (TWGHs) Even Centre in Hong Kong SAR for their issues related to their family member’s gambling problems within the data collection period (March 2011 to February 2012) were invited to join the study. Inclusion criteria were being over the age of 18 years, of Chinese ethnicity and able to speak and read Chinese. The exclusion criteria included manifestation of signs of cognitive impairments and/or showing imminent suicidal risk assessed and reported by their counsellors. In order to capture diverse experiences and avoid overlapping responses, when there was more than one family member from the same family presented at the service, the family would be asked to nominate one representative for this study. A total of 103 family members successfully completed the questionnaire. The response rate was 60 %.

Ethics approval was granted by the University of Melbourne’s Humanities and Applied Sciences Human Ethics Sub-Committee (Ethics ID: 0830146) and the Victorian Department of Justice Human Research Ethics Committee (CF11/19644). The data were collected by trained professionals such as social workers and researchers. Consent was obtained from all respondents in the study.

### Measures

The questionnaire included questions on demographic information (including gender, age, relationship status, highest educational qualification, living status, employment status, yearly income, and relationship to the gambler), health and psychological well-being, gambling-related family member impacts, and family coping. All questions included in the demographics and selected original English measurements were translated from English into traditional Chinese and back translated. The final questionnaire was piloted with a small number of family member service users to ensure clarity of the translation.

### Health and psychological well-being

*Kessler Psychological Distress Scale (K10)* (Kessler & Mroczek, 1994). The K10 is a validated measure of current (1 month) non-specific psychological distress. The scale comprises 10 questions asking respondents to indicate how frequently they experienced specific symptoms of psychological distress, such as nervousness, agitation, psychological fatigue and depression. Each item has a 5-point response format ranging from (1) none of the time to (5) all of the time and the item scores are summed to obtain a total score. Scores can be categorised as low (10–15), moderate (16–21), high (22–29), and very high (30–50). The K10 has displayed very good agreement with the World Health Organization Composite International Diagnostic Interview module for mood and anxiety disorders (Furukawa, Kessler, Slade & Andrews 2003) and serious mental illness (Kessler, Barker, Epstein et al., 2003), with areas under the curve (AUC) ranging from 0.854 to 0.929. Reliability analyses in this study showed that the scale was very reliable in the current sample, with Cronbach alphas 0.91 and mean inter-item correlations greater than 0.51.

*Short Form General Health Survey*—*single item (SF*-*1)* (Ware, Snow, Kosinski & Gandek, 1993). General health was assessed by using the SF-1, a single item measure of general health derived from the SF-36 (Ware et al., 1993). Family members were asked to report on their own health condition by the question: ‘In general, how would you describe your health?’ The response options ranged from (1) poor to (5) excellent. The SF1 is widely used in routine health surveys and has excellent psychometric qualities for a single item measure.

*World Health Organisation*-*Quality of Life BREF* (Murphy, Herrman, Hawthorne, Pinzone & Evert, 2000). Quality of life was measured using the first item of the WHOQOL-BREF (Australian version). Family members were asked to answer a question about their perceived overall quality of life: ‘How would you rate your quality of life?’ The response options ranged from (1) very poor to (5) very good. Test–retest reliability in an Australian sample (n = 114) is .57. Discriminant validity of the single item is good as it discriminates well across the full health spectrum (Murphy et al., 2000).

### Gambling-related Family Member Impacts

*Family Member Impact (FMI) Scale* (Orford et al., 2005). The 16-item FMI scale was originally designed to measure the extent and type of harmful impacts on the family members of individuals with drinking or drug use problems. It has since been adapted for use with family members of relatives with gambling problems (Orford et al., 2005). Family members were asked to report the impact of problem gambling on themselves. Each item is followed by a four-point response option (0 = not at all, 1 = once or twice, 2 = sometimes, 3 = often). The scale scores were summed to produce a total family impact score, and were also categorized to give two subscale scores reflecting two different aspects of impact on family: (1) Worrying Behaviour (items related to the level of worry that the family members feel they have with regard to the client); and (2) Active Disturbance (items relating to physical disturbances caused by the problem gambler relative, together with difficulties due to the gambling problems). The FMI subscales demonstrated moderate to good internal consistency with Cronbach alpha 0.82 for Worrying Behaviour Subscale, and 0.85 for Active Disturbance Subscale in the current sample.

### Gambling related family coping

*Coping Questionnaire (CQ)* (Orford, Guthrie, Nicholis et al., 1975) The CQ was originally devised to be administered to wives of men with drinking problems, and it has been widely used in the United Kingdom and various countries (Hayashi, 1978; Holmila, 1997; McCrady & Hay, 1987). The version of the 30-item CQ used in the present study was adapted for family members for gambling problems (Krishnan & Orford, 2002; Orford et al., 2005). It aims to measure the frequency of coping actions in response to a family member’s excessive gambling behaviour. The scale comprises three subscales: (1) Engaged Coping Response (engaging in trying to change a relative’s excessive gambling in a variety of ways that may be emotional, assertive, controlling and/or supportive); (2) Tolerant-inactive Coping Response (putting up with a relative’s gambling, involving accepting it, making sacrifices in the face of it or encouraging it); and (3) Withdrawal Coping Response (withdrawing from the relative or engaging in activities independently of the relative). The CQ scales display good internal reliability (α = 0.60–0.85) (Orford et al., 2005) and discriminant validity (Krishnan & Orford, 2002). Family members were asked to report their own coping responses as a result of the problem gambling in the 12-months prior to the interview. In the current study, results showed that the internal consistency for all the subscales were acceptable to good (Cronbach alpha of 0.83 for Engaged Coping; Cronbach alpha of 0.75 for Tolerant-inactive Coping; Cronbach alpha of 0.59 for Withdrawal Coping).

### Data analysis

Missing data were coded to ensure that the incomplete data records could be detected easily. Participants with any missing data in a particular scale or subscale were deleted for that scale or subscale. Descriptive statistics were performed for aims 1–3. Non-parametric Spearman’s correlation analyses were used for the fourth aim to determine whether gambling-related family coping strategies relate to the impact of gambling on the family since both subscales of the FMI, K10 and CQ were non-normally distributed (with the exception of the Worrying Behaviour subscale of the FMI). A series of cross-tabulations were also conducted to understand the distribution of demographic characteristics (e.g. age, gender, income and employment) on health and psychological wellbeing, impacts, and coping strategies.

## Results

### Demographic characteristics

The majority of family members were female (87.0 %, n = 87). The mean age of participants was 44.6 years (SD = 11.9), with the majority of family members aged 41–50 years (29.1 %, n = 30) followed by 51–60 years (23.3 %, n = 25) and 31–40 years (19.4 %, n = 20). Most family members reported being married or cohabitating (70.8 %, n = 73), followed by being never married (15.5 %, n = 16) or divorced (3.9 %, n = 4). Most family members attained high school education (49.5 %, n = 51), followed by primary school or below (27.2 %, n = 28). The majority (85.4 %, n = 88) of family members reported living with other family members, with a smaller proportion living as couple (8.7 %, n = 9) or living alone (3.9 %, n = 4). Most family members worked full-time or part-time (68.9 %, n = 71) or were homemakers (22.3 %, n = 23), with a smaller proportion of retired participants (7.8 %, n = 8). Most family members revealed having low or no income. One-third (30.1 %, n = 31) reported having income ranging from HK$5001 to HK$10,000 per month followed by no income (23.3 %, n = 24) and having income ranging from HK$1 to HK$5000 (16.5 %, n = 17). Most of the family members were spouses, partners or ex-partners of the gamblers (63.1 %, n = 65), followed by offspring (17.5 %, n = 18) and siblings (13.6 %, n = 14).

### Health and psychological wellbeing

Family members were invited to report their level of psychological distress on the K10. The mean for psychological distress was 20.2 (SD = 7.3), with most (61.8 %) reporting moderate psychological distress. Over one-quarter (27.4 %) of family members reported low or no psychological distress, followed by high psychological distress (10.8 %). According to the result on the single-item general health condition question, the majority of family members (51.5 %, n = 53) reported fair health followed by good (32.2 %, n = 33) and poor (8.7 %, n = 9) health. Only 3.9 % (n = 4) reported having good or excellent general health. The mean score for the general health condition for the family members was 2.4 (SD = 0.9). Most of the family members reported neither good nor bad quality of life (54.4 %, n = 56) followed by good (30.1 %, n = 31) and poor (9.7 %, n = 10) quality of life on the single-item Quality of Life—WHO BREF question. The mean score on Quality of Life—WHO BREF question for family members was 3.3 (SD = 0.8). A series of cross-tabulations to understand the distribution of demographic characteristics on health and psychological wellbeing revealed no significant findings.

### The impact of gambling on the family

Family members were invited to report on the Family Member Impact Scale about the impact of gambling on themselves. The mean scores for the Worrying Behaviour subscale and the Active Disturbance subscales were 17.0 (SD = 7.2) and 8.1 (SD = 4.3) respectively. The scores for the Worrying Behaviour subscale and the Active Disturbance subscale were significantly lower than those based on a sample of family members of relatives with alcohol, drugs or gambling problems in a previous study (Worrying Behaviour: Mean = 19.92, t(101) = −4.02, p < 0.001; Active Disturbance: Mean = 10.71, t(101) = −6.23, p < 0.001) (Orford et al. 2005). A series of cross-tabulations to understand the distribution of demographic characteristics on family impacts revealed no significant findings.

### Gambling-related family coping

Family members were invited to report on their own gambling related coping among the three kinds of coping responses: engaged, tolerant-inactive and withdrawal. The mean scores for Engaged Coping, Tolerant-inactive Coping and Withdrawal Coping subscale were 23.7 (SD = 8.2), 8.9 (SD = 5.4) and 10.4 (SD = 4.3) respectively. The scores for the Engaged Coping subscale, the Tolerant-inactive Coping subscale and the Withdrawal Coping subscale were significantly higher than the previous sample of family members of relatives with gambling problems that was used in the development of the scale (Engaged Coping: Mean = 21.7, t(102) = 2.44, p < 0.05; Tolerant-inactive Coping: Mean = 5.2, t(100) = 6.98, p < 0.001; Withdrawal Coping: Mean = 6.2, t(99) = 9.58, p < 0.001) (Krishnan & Orford, 2002). A series of cross-tabulations to understand the distribution of demographic characteristics on family coping revealed no significant findings.

### Correlations between impact of gambling on the family, psychological distress and family coping strategies

There were significant positive correlations between the Worrying Behaviour subscale of Family Member Impact (FMI-WB) and Engaged Coping (CQ-E), Tolerant-inactive Coping (CQ-T) and Withdrawal Coping (CQ-W) subscales of CQ. The results also showed strong correlations between Active Disturbance subscale of Family Member Impact (FMI-AD) and all subscales of CQ. Significant positive correlations between FMI-WB, FMI-AD and psychological distress (K10) were also found. K10 was also significantly correlated with the subscales of CQ (CQ-E and CQ-T) except CQ-W (Table 1).

**Table 1:** Correlations among the impact of gambling on family, psychological distress, and gambling related family coping

	CQ-E	CQ-T	CQ-W	FMI-WB	FMI-AD
K10	0.44***	0.51***	0.19	0.53***	0.54***
CQ-E		0.70***	0.18	0.49***	0.55***
CQ-T			0.96	0.49***	0.55***
CQ-W				0.45***	0.52***
FMI-WB					0.79***

*** p < .001; ** p < .01; * p < .05

K10—Kessler 10 Scale; CQ-E—Coping Questionnaire–Engaged Coping Subscale; CQ-T—Coping Questionnaire–Tolerant-inactive Coping Subscale; CQ-W—Coping Questionnaire–Withdrawal Coping Subscale; FMI-WB—Worrying Behavior Subscale of Family Members Impact; FMI-AD—Active Disturbance Subscale of Family Members Impact

## Discussion

This study has generated original and valuable data for understanding the impacts of problem gambling on family members and the relationship between family coping and gambling-related family impacts in Chinese family members of problem gamblers from Hong Kong. The findings of this study may help to generate culturally appropriate theoretical models in facilitating the development of family sensitive and effective treatment programs.

In this study, the family member samples were comprised mostly of help-seeking family members who were female partners or ex-partners of gamblers. Since there is evidence that other extended family members and concerned significant others (CSOs) such as siblings, parents, grandparents, friends and colleagues are also affected by problematic gambling behaviour (Dowling et al., 2014a), further investigation is needed to understand more about the impacts on CSOs other than partners in the Chinese communities.

Most family members (61.8 %) reported a medium level of psychological distress with an additional 10.8 % reporting a high level. Most family members (60.2 %) reported fair or poor health on the single-item General Health question and 64.1 % reported poor or neither good nor bad quality of life using the single-item Quality of Life question. These results correspond with previous studies showing that affected family members often suffer from a wide range of health and psychological difficulties (Dowling et al., 2014a; 2015; Lesieur, 1998; Lorenz & Shuttlesworth, 1983; Lorenz & Yaffee, 1988; Orford et al., 2013; Wenzel et al., 2009).

Although the scores for Family Member Impacts were lower than the family members of individuals with addictions (alcohol, drug or gambling problems) from a previous study (Orford et al., 2005), the levels of coping were significantly higher than those from the previous small sample of family members of problem gamblers (Krishnan & Orford, 2002). These findings may be due to cultural differences in the way Chinese family members experience impacts of gambling, and the way they adopt coping strategies. They may, however, also be due to the clinical and convenience sampling method employed in the current study, whereby clinical samples may initiate positive coping through help seeking decisions. Future research exploring the differences in family impact and coping of gambling disorder between clinical and non-clinical populations within the Chinese communities may generate a clearer understanding of how family members cope in the face of a relative’s gambling disorder. For the current results, we can assume that for family members who employed positive coping through help seeking actions might reduce the level of adverse impacts on themselves. This reinforces the need for public education on reinforcement of help seeking and positive coping behaviours for family members affected by gambling disorder.

The findings of the current study indicate that among the three identified coping styles, tolerant-inactive coping seems to have the strongest correlation with psychological distress. Lee, Manning, Winslow et al., (2011) reported that family members of relatives with addiction displayed significantly greater depression, stress and psychiatric morbidity and poorer overall well-being. This study also found that among the three coping responses, tolerant-inactive coping was the most strongly correlated with psychological well-being subscales (Lee et al., 2011). It has been reported that people adopt different kinds of coping strategies when distress becomes more severe and that people tend to move from self-help to seeking professional help when distress intensifies (Jorm, Griffiths, Christensen et al., 2004). These findings may, however, be influenced by cultural characteristics. For example, endurance has been identified as one of the primary coping strategies which emphasize avoidance of direct confrontation by family members that could increase the likelihood of enlisting third party intervention. This form of coping however might create addition stress for family members (Mokuau, 1991). Future studies on factors contributing to the coping styles adopted by Chinese family members would be meaningful in treatment planning.

No significant relationship was found between withdrawal coping and psychological distress. This finding was consistent with the previous findings that ways of coping influence symptoms of strain (i.e., that not all forms of coping are correlated with strain) (Orford et al., 2005). These findings suggest that family members who adopt withdrawal coping tend to withdraw themselves emotionally from the stressful situation and are therefore not as affected as others who adopt engaged coping and tolerant-inactive coping. This interpretation is consistent with the current study’s results that tolerant-inactive coping displayed the strongest relationship with family member impacts and psychological distress followed by engaged coping and withdrawal coping. Family members who adopt withdrawal coping ostensibly minimize adverse impacts on themselves by disconnecting emotionally from the gambler and the situation.

The correlation findings in the current study therefore support aspects of the stress–strain–coping model that family member impact (stress) is positively correlated with psychological distress (strain) and family coping (coping). It is presumed from these findings that more coping strategies are employed by family members when the impacts of gambling are high. It is common for people to apply more coping responses when they are faced with painful and threatening events. Since the correlational analyses did not include causal relationships, it is reasonable to assume that a more stressful situation will stimulate more coping responses (Orford, Natera, Velleman et al., 2001). Further research is required to test the degree to which coping and social support received by the family members of problem gambling play a role in the stress–strain relationship via buffering, mediational, or additive effects (Orford et al., 2005).

### Limitations

There are a number of limitations to this study. The current study employed clinical and convenience samples from a treatment service. While positive coping may be initiated through help seeking decisions, treatment-seeking family members are likely to have experienced higher impacts as a result of the family member’s gambling behaviour. Hence, a bias created by the treatment-seeking nature of the sample cannot be excluded. This may also affect the sensitivity of the selected scales. Second, a relatively small sample size in the current study may limit the validity of the analysis. Third, a gender difference was obvious in the current sample in that most (87 %) family members were female, with only 13 % being male. This gender bias may generate useful gender-specific data but may affect the generalisability of the results.

### Recommendations

The current study has generated findings and insights that have implications for practice and future research. Since the aim of this paper is to look at the relationships between family members’ impacts, psychological distress and coping (stress–strain–coping), future analysis to explore how coping strategies (CQ) mediate or moderate the relationship between stress and strain would be meaningful to further our understanding and develop effective programs for family members. In terms of treatment modality, it is recommended that skills enhancement programs and treatment groups in family functioning and family coping be developed. Although there are some family focused coping programs in the Western cultures, clinical trials are needed for Chinese cultures. Culturally sensitive treatment programs with consideration of specific cultural beliefs should be emphasised. Treatment components should involve adaptation of healthy coping strategies including self-care, interests building and expansion of social support.

It is also suggested that development and validation of a culturally sensitive screening protocol for gambling-related family impacts and family coping will contribute to the design of an integrated and family sensitive service model and case management plan. Since problem gamblers tend not to seek help until they have reached a crisis, an increase in public health promotion to encourage family members’ help seeking as early as possible is recommended. Finally, it is vital to conduct professional training to gambling counsellors and other health care professionals to enhance their competency in assessing and treating family members who are affected by problem gambling.
